# Challenges of rabies surveillance in Madagascar based on a mixed method survey amongst veterinary health officers

**DOI:** 10.3389/fvets.2024.1270547

**Published:** 2024-02-28

**Authors:** Anou Dreyfus, Marie Hermelienne Volasoa, Hélène Guis, Nivohanitra Perle Razafindraibe, Mino Harimbola Razafindramparany, Nomenjanahary Lalaina Arivony, Naltiana Rakotondrabe, Mamitiana Aimé Andriamananjara, Philippe Dussart, Daouda Kassie, Vincent Lacoste, Soa Fy Andriamandimby

**Affiliations:** ^1^Unité d’Epidémiologie et de Recherche Clinique, Institut Pasteur de Madagascar, Antananarivo, Madagascar; ^2^Unité de Virologie, Institut Pasteur de Madagascar, Antananarivo, Madagascar; ^3^Département des Enseignements des Sciences et de Médecine Vétérinaire, Université d’Antananarivo, Antananarivo, Madagascar; ^4^CIRAD, UMR ASTRE, Antananarivo, Madagascar; ^5^Astre, Université de Montpellier, CIRAD, INRAE, Montpellier, France; ^6^Epidemiology and Public Health Unit, Institut Pasteur du Cambodge, Phnom Penh, Cambodia; ^7^Directorate of Veterinary Services, Ministry of Agriculture and Livestock, Antananarivo, Madagascar

**Keywords:** rabies, surveillance, survey, veterinary health officer, Madagascar, dog-mediated rabies, zoonosis, one health surveillance

## Abstract

**Introduction:**

In Madagascar, rabies is endemic and a notifiable disease. The objective of this mixed study was to understand the challenges faced by the Veterinary Health Officers (VHOs) in the current rabies surveillance system in Madagascar.

**Methods:**

A survey was conducted from mid-April to the end of July 2021 during which all officially-listed VHOs (*N* = 150) were contacted by phone at least twice. Participants, representative of the 22 regions of Madagascar, were interviewed by phone based on a semi-structured questionnaire on (1) their knowledge of rabies epidemiology in their area of activities, (2) the occurrence of human and animal rabies and the species affected in the region where they work, (3) the factors that might influence rabies surveillance depending on (a) their activities and area of operation, (b) the socio-cultural aspects of local communities, and (c) the general organization of animal rabies surveillance.

**Results:**

The majority (80/90) of VHOs declared having been informed of at least one suspected or confirmed case of human and/or animal rabies in their area of activity during their work as VHOs: at least once a month for 11 of them, at least once a year for 40 and with undetermined frequency for 29. Several obstacles hinder the surveillance of rabies resulting in under-reporting. The lack of funds to access suspect animals, collect, pack and ship samples in compliance with biosecurity measures and the cold chain, was mentioned as a major obstacle to surveillance. The second barrier is socio-cultural: in many large coastal regions, dogs are taboo and VHOs fear rejection by the community if they treat dogs.

**Discussion and conclusion:**

While the general population requires information on the rabies situation in Madagascar and on how to proceed in the event of a bite, veterinarians and decision-makers need to be fully aware of an evidence-based approach to rabies surveillance, prevention and control. Communication between the human and animal health sectors should be improved. Politicians need to be persuaded of the importance of funding to eliminate rabies in Madagascar. The adoption, in early 2023, of a national strategic plan for rabies control is a first step in this direction.

## 1 Introduction

Rabies is a fatal viral zoonosis transmitted to humans by dogs in 99% of cases. It is responsible for approximately 60,000 human deaths per year, mainly in Asia and Africa (1). In Madagascar, rabies is endemic and a notifiable disease in humans and animals. Surveillance is based on a veterinary/medical (suspect cases) and laboratory (confirmed cases) reporting system (2). The National Reference Laboratory (NRL) for rabies hosted by the Virology Unit of the Institut Pasteur de Madagascar (IPM), carries out rabies diagnosis for free. Samples from suspected animal and human rabies cases are sent to the NRL, which then notifies all confirmed cases to both the Veterinary Services of the Ministry of Agriculture and Livestock, and to the Ministry of Public Health.

In Madagascar, most dogs, with or without owners, roam freely in the streets. Rabies vaccination coverage in dogs is very low as very few vaccination campaigns have been performed by the Ministry of Agriculture and Livestock or by private or non-governmental organizations (NGO) in recent years. In addition, most dogs are never seen by a veterinarian due to access difficulties, cultural issues (see below), education and costs. In this context, published data on vaccination coverage are scarce and usually limited to a given city or district (3–5). A study conducted in 2007–2008 in the capital city of Antananarivo on 2,180 owned dogs showed that the percentage of regularly vaccinated dogs with a valid vaccination certificate was 7.2% (95% CI 6.2–8.4%) (3). Ten years later, another study, conducted in the rural commune of Andasibe revealed that only 5% of dogs had a history of vaccination (4). However, in 2018, the vaccination coverage was high (62.5%) in Moramanga, a medium-sized city, due to a vaccination campaign carried out by an NGO called “Mad Dog Initiative” but was extremely low (2.4%) in surrounding rural communes (5).

Thirty-one anti-rabies treatment centers (ARTC) are spread over the 22 regions of Madagascar where an average of 15,000 bitten or scratched human patients receive post-exposure prophylaxis (PEP) each year (6). Rajeev et al. (7) estimated 960 (95% Prediction Intervals (PI): 790–1,120) human deaths from rabies annually, with PEP preventing an additional 800 (95% PI: 640–970) deaths. Given the paucity of data, rabies deaths were estimated as a function of the number of reported dog bites predicted by a Poisson regression model accounting for the distance to PEP health centers and estimates of the incidence of exposure to endemic rabies using an adapted decision tree framework. Exposure incidence data originated from the Moramanga district (42 exposures/100,000 persons) and assumed a 1% rabies incidence in dogs (8).

From 2011 to 2020, the annual number of animal samples sent to the NRL ranged from 55 (in 2019 and 2020) to 151 (in 2012). The proportion of rabies confirmed samples ranged from 56% (95% CI 45.2–66.7) in 2013 to 78% (95% CI 45.8–76.2) in 2012 (9). Given the low number of samples received, the high percentage of rabies confirmed cases and the limited number of districts sending samples (*n* = 12 to 23 out of 114 per year), it is very likely that we are only seeing “the tip of the iceberg” and that underreporting is frequent, as in most low-income countries where canine rabies is endemic (10). Furthermore, samples of rabies-suspect animals received by the NRL were geographically-clustered. Indeed, 74.8% (*n* = 383/512) of those of known origin, received between 2010 and 2015, were from the Analamanga region, which includes the capital Antananarivo (11). The NRL data are thus not representative of the entire country. At last, the NRL database indicates that, over the past 10 years, a larger number of samples (66.7%) were sent to the NRL by citizens (mostly dog owners) rather than by veterinary health officers (VHOs) (9), who are officially responsible for reporting and controlling suspected rabies cases in the animal population.

Madagascar has many different ethnic groups, mainly of African and Asian ancestry. Each group generally lives in a limited geographical area, covering one or a few districts. Some of these ethnic groups, located in the Western, Southern and Eastern coastal regions, consider dogs as “fady,” a Malagasy term meaning “taboo.” One example is the Antemoro (or Antaimoro) people who live on the southeastern coast, mostly between Manakara and Farafangana.^1^ Consequently, for many people in these regions, touching and caring for dogs goes against their cultural beliefs, which represents a challenge for rabies surveillance and control (12, 13).

The objective of this mixed method study was therefore to understand the challenges faced by VHOs in the current rabies surveillance system in Madagascar. The survey’s objectives were to (1) evaluate their knowledge of rabies epidemiology, (2) describe the occurrence of human and animal rabies in their work area, (3) determine the factors that might influence rabies surveillance depending on (a) their activities/roles and area of operation, (b) socio-cultural aspects of local communities, and (c) the overall organization of animal rabies surveillance and (4) compare occurrence of rabies reported by VHOs to data from the NRL to map what is currently known on rabies circulation at the district level.

## 2 Methods

### 2.1 Study population

VHOs are private veterinarians mandated by the government to carry out various public health activities in accordance with their legal, technical and territorial competence. The veterinary mandate is issued by order of the Ministry of Agriculture and Livestock, which oversees animal health. Their activities consist of providing collective prophylaxis for animals in their area of jurisdiction (collective vaccination, deworming, testing for animal diseases, collective treatment, issuing of vaccination or treatment certificates), as well as undertaking epidemiological surveillance of animal diseases, sanitary control and inspection related to veterinary public health (in particular meat hygiene).

### 2.2 Data collection

The survey was conducted from mid-April to the end of July 2021. A comprehensive list of VHOs was obtained from the Veterinary Services, Ministry of Agriculture and Livestock. All VHOs were contacted by phone. Those, who were not reached the first time were called back immediately and then after 2 months. The participating VHOs were interviewed by phone in Malagasy language based on a semi-structured questionnaire with open and closed questions (Supplementary Information). The KoboToolbox survey platform was used for data entry. Interviews were recorded and completed forms were exported to an online database at the end of each interview.

### 2.3 Data analysis

All responses to the closed questions were exported to an excel database and frequencies were calculated in R® and in Excel®. Responses to the open questions were transcribed and a thematic analysis of the textual data was carried out by developing a thematic analysis grid in which responses were grouped into subcategories for subsequent statistical analysis. All data were analyzed in R® version 4.0.4 and Excel®.

### 2.4 Ethical considerations

Oral consent to participate in this survey was obtained from the respondents at the beginning of the phone call. Study participants were informed at the beginning of the interview of the purpose of the study and that the interview was recorded but that the data would be used anonymously. They were also informed of their right to refrain from answering a question or to withdraw their participation at any time.

## 3 Results

### 3.1 Study population and region

**Figure 1:**
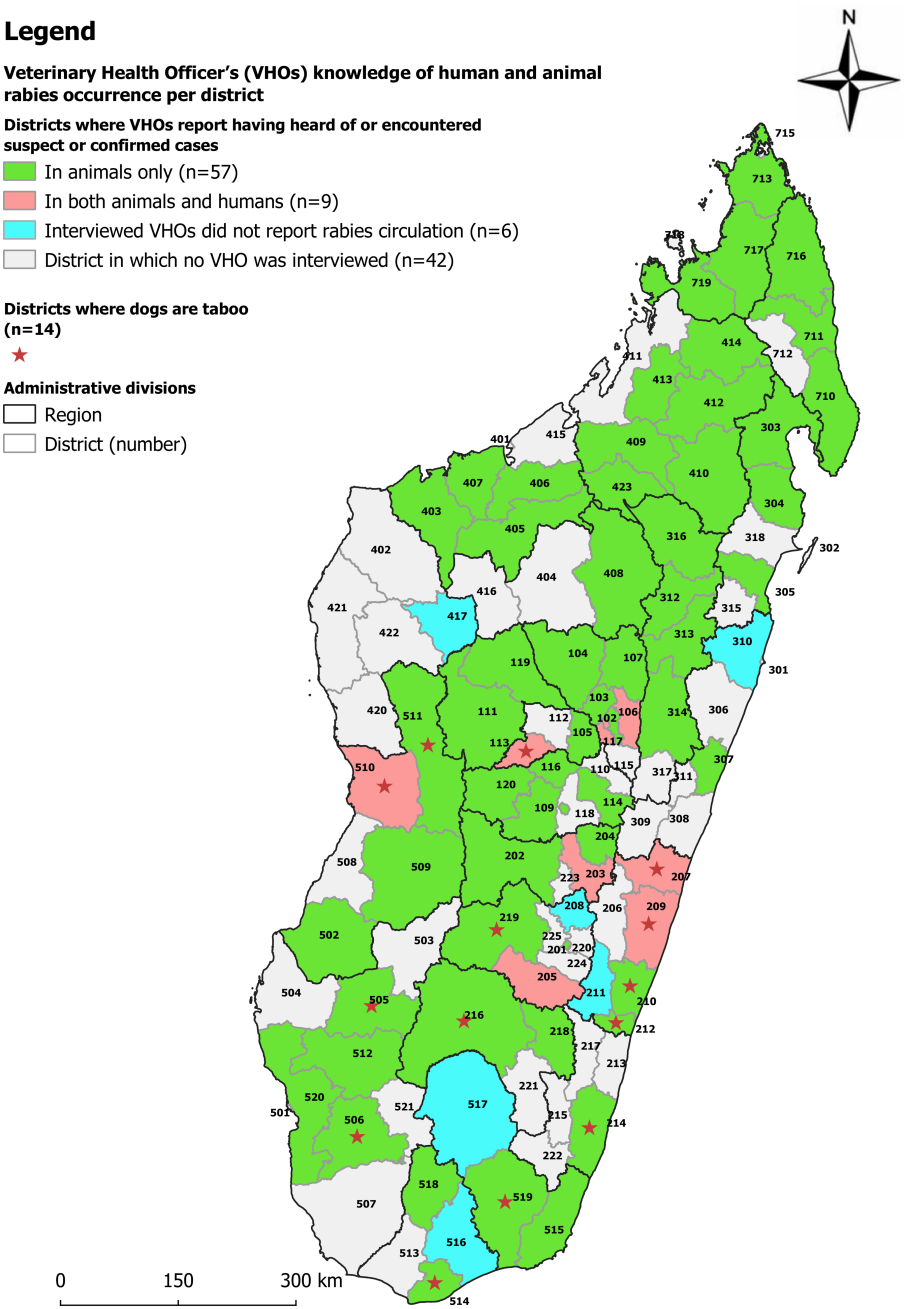
Map of Madagascar divided into regions and districts, illustrating the Veterinary Health Officers (VHOs) knowledge on occurrence of human and/or animal rabies cases. The name of the districts is listed by the number in Table 1 and Supplementary Figure 1.

Madagascar is geographically divided into districts (*N* = 114) and regions (*N* = 22) (Figure 1). We contacted 150 VHOs by phone of which 90 agreed to participate in the survey. Participating VHOs were from the 22 regions of Madagascar and 72 of the 114 districts. Of the 60 VHOs from 42 districts who did not take part in the survey, two declined to participate and 58 were unable to respond. The reasons for not responding were not having a network connection at the time of the calls (phone switched off or outside of connection signal) (*n* = 31); not answering the calls, despite having been contacted twice (*n* = 26); or having deceased (*n* = 1). Interviewed VHOs had been working in their area for at least two years at the time of the survey (2 to 31 years, mean = 17.2 (95% CI 15.3–19.1)). Their offices are located along the main national roads, but they collaborate with veterinary assistants or communal animal health workers who provide veterinary assistance/service in very remote areas. Most VHOs (79%) reported having a work radius of more than 25 km. Their working area can cover several districts and some VHOs have overlapping working districts.

### 3.2 Types of clients

Most VHOs reported to work primarily with livestock (cattle, pigs) (95%; 86/90) and poultry (88%, 79/90), and to rarely treat “pets,” such as dogs. Twenty-two VHOs (24.4%) reported not treating dogs at all for socio-cultural reasons, either because they were worried losing their clientele if they treated dogs (*n* = 9), or even out of personal conviction (*n* = 4). This is because some ethnic groups in several districts of Madagascar consider dogs to be a taboo animal, not to be touched or cared for. More detailed information can be found in section 3.5.

### 3.3 Knowledge of rabies epidemiology in their area of activities

During the interview, we assessed the VHOs knowledge of rabies epidemiology, transmission and vectors. While the role of dogs in rabies transmission was unanimously known, 80% (72/90) of the VHOs stated that the main vector of rabies in their locality was stray dogs and 15% (14/90) suspected that hungry feral dogs attacking livestock were the main vector.

### 3.4 Occurrence of rabies

The majority (80/90) of VHOs declared having encountered or been informed of at least one suspected or confirmed case of human and/or animal rabies in their area of activity during their work as VHOs. Overall, 89% (80/90) of VHOs reported human or animal rabies from 92% (66/72) of districts distributed among the 22 regions. Nine (10%) VHOs reported the occurrence of human cases in nine (12%) districts (numbers 101, 106, 113, 117, 203, 205, 207, 209, and 510 in Figure 1) (see Table 1 and Supplementary Figure 1 for the names of the regions). The ten (11%) VHOs that reported not having heard or observed any animal or human rabies circulation were from six districts illustrated in Figure 1 by numbers 417, 208, 516, 517, 211 and 310.

**Table 1:** Districts of Madagascar per region, which can be located on the maps of Figures 1–3 by referring to the number.

Districts of Tananarivo Province		Districts of Fianarantsoa Province		Districts of Toamasina Province		Districts of Mahajanga Province		Districts of Toliary Province		Districts of Antsiranana Province	
Number	Name	Number	Name	Number	Name	Number	Name	Number	Name	Number	Name
Region ANALAMANGA		Region HAUTE MATSIATRA		Region ATSINANANA		Region BOENY		Region ATSIMO ANDREFANA		Region SAVA	
101	Antananarivo Renivohitra	201	Fianarantsoa I	301	Toamasina I	401	Mahajanga I	501	Toliary-I	710	Antalaha
102	Antananarivo Avaradrano	205	Ambalavao	306	Brickaville	403	Soalala	503	Beroroha	711	Sambava
103	Ambohidratrimo	208	Ambohimahasoa	307	Vatomandry	405	Ambato Boeni	504	Morombe	712	Andapa
104	Ankazobe	219	Ikalamavony	308	Mahanoro	406	Marovoay	505	Ankazoabo	716	Vohemar
106	Manjakandriana	220	Lalangina	309	Marolambo	407	Mitsinjo	506	Betioky Atsimo		
107	Anjozorobe	224	Vohibato	310	Toamasina II	415	Mahajanga II	507	Ampanihy Ouest	***Region DIANA***	
115	Andramasina	225	Isandra	311	Antanambao Manampontsy			512	Sakaraha	713	Antsiranana II
117	Antananarivo Atsimondrano					***Region MELAKY***		520	Toliary-II	715	Antsiranana I
		***Region AMORON I MANIA***		***Region ANALANJIROFO***		402	Besalampy	521	Benenitra	717	Ambilobe
***Region ITASY***		202	Ambatofinandrahana	302	Sainte Marie	417	Ambatomainty			718	Nosy-Be
105	Arivonimamo	203	Ambositra	303	Maroantsetra	420	Antsalova	***Region MENABE***		719	Ambanja
112	Miarinarivo	204	Fandriana	304	Mananara-Avaratra	421	Maintirano	502	Manja		
113	Soavinandriana	223	Manandriana	305	Fenerive Est	422	Morafenobe	508	Morondava		
				315	Vavatenina			509	Mahabo		
***Region VAKINANKARATRA***		***Region VATOVAVY FITOVINANY***		318	Soanierana Ivongo	***Region BETSIBOKA***		510	Belo Sur Tsiribihina		
108	Antsirabe I	206	Ifanadiana			404	Maevatanana	511	Miandrivazo		
109	Betafo	207	Nosy-Varika	***Region ALAOTRA MANGORO***		408	Tsaratanana				
110	Ambatolampy	209	Mananjary	312	Amparafaravola	416	Kandreho	***Region ANDROY***			
114	Antanifotsy	210	Manakara Atsimo	313	Ambatondrazaka			513	Beloha		
116	Faratsiho	211	Ikongo	314	Moramanga	***Region SOFIA***		514	Tsihombe		
118	Antsirabe II	212	Vohipeno	316	Andilamena	409	Port-Berge (Boriziny-Vaovao)	516	Ambovombe-Androy		
120	Mandoto			317	Anosibe-An’ala	410	Mandritsara	518	Bekily		
		***Region IHOROMBE***				411	Analalava				
***Region BONGOLAVA***		216	Ihosy			412	Befandriana Nord	***Region ANOSY***			
111	Tsiroanomandidy	218	Ivohibe			413	Antsohihy	515	Taolagnaro		
119	Fenoarivobe	221	Iakora			414	Bealanana	517	Betroka		
						423	Mampikony	519	Amboasary-Atsimo		
		***Region ATSIMO ATSINANANA***									
		213	Farafangana								
		214	Vangaindrano								
		215	Midongy-Atsimo								
		217	Vondrozo								
		222	Befotaka								

**Figure 2:**
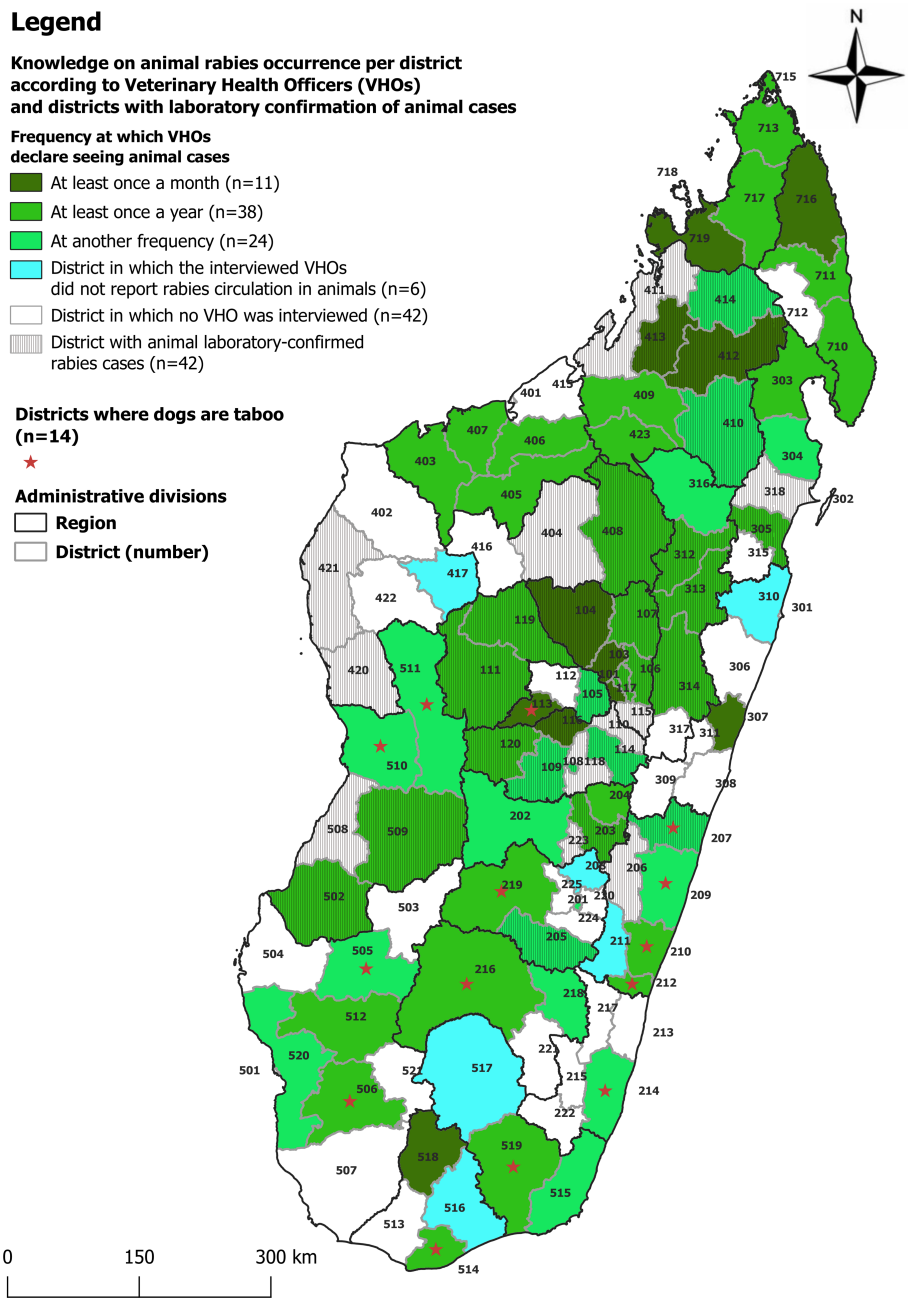
Map of Madagascar divided into regions and districts, illustrating the Veterinary Health Officers (VHOs) knowledge on the frequency of animal rabies occurrence and districts with confirmed animal cases by the National Reference Laboratory for Rabies at the Institut Pasteur de Madagascar. The name of the districts is listed by the number in Table 1 and Supplementary Figure 1.

In terms of frequency, 11 (12%) VHOs from 11 (15%) districts said that they had heard of rabies cases in their area at least once a month, 40 (44%) from 38 (53%) districts at least once a year and 29 (32%) from 18 (25%) districts at an undetermined frequency (Figure 2). For those who had their mandate for more than 10 years (64 VHOs), 9 said that they had heard of rabies cases in their area at least once a month, 31 at least once a year and 18 at an undetermined frequency. Most of these suspected or confirmed cases concerned dogs (80 VHOs), bovines (18 VHOs) or humans (9 VHOs) and 9 VHOs mentioned cases linked to other species (small ruminants or pigs).

The reasons stated by the VHOs for the occurrence of human rabies cases were negligence/unawareness of the danger of dog bites (*N* = 18). Sixteen VHOs stated that even in case of a dog bite, victims and even medical doctors take no specific action. Another reason stated was the difficulty of accessing ARTCs, which offer PEP. These comments were made during the free discussion (and collected in the analysis grid) and not all VHOs were asked the question systematically. Absence of dog vaccination was not mentioned.

#### 3.4.1 Comparison of VHOs knowledge on rabies occurrence and NRL confirmed rabies cases

**Figure 3:**
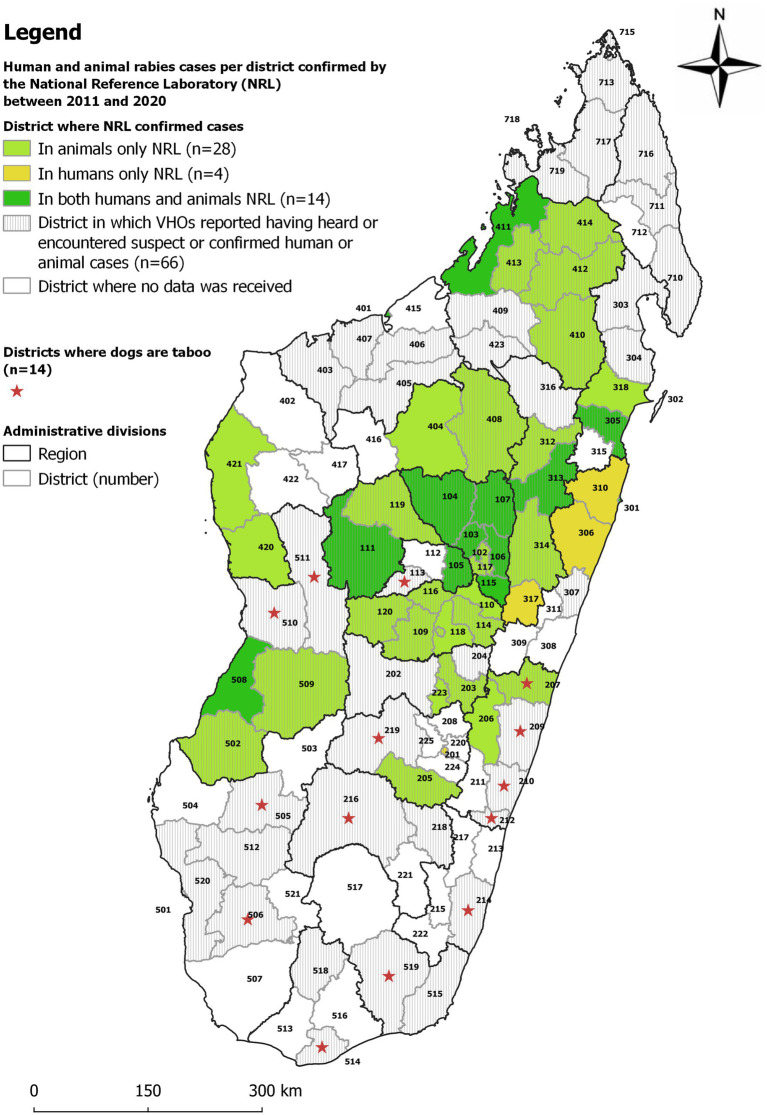
Map comparing districts with confirmed human or animal rabies cases from 2011 to 2020 by the National Reference Laboratory for Rabies at the Institut Pasteur de Madagascar and VHO-reported rabies occurrence. The name of the districts is listed by the number in Table 1 and Supplementary Figure 1.

Data on human or animal rabies cases confirmed by the NRL from 2011 to 2020 are presented in Figure 3. Confirmed human and animal cases were registered by the NRL in 18 (16%) and 42 (37%) districts, respectively (in 14 districts both confirmed human and animal cases were recorded). The areas without any confirmed cases (either because (i) no sample was received, (ii) sample was too deteriorated to be tested or (iii) no sample tested positive) are located in the five most southern regions of Madagascar (Atsimo Andrefana, Androy, Anosy, Ihorombe, Atsimo Atsinanana), the two most northern ones (Diana, Sava) and Boeny (north west) region. From 13 of the 14 districts where dogs are considered taboo by many persons (see Figure 1 and section 3.5 on this taboo), no confirmed cases were registered by the NRL.

NRL data was compared with the data collected through interviews of VHOs (Table 1; Figures 1–3; Supplementary Figures 2, 3). Overall, when combining the declarations made by the VHOs and the results of confirmed cases registered by the NRL from 2011 to 2020, animal and human rabies were reported to be circulating in 79 and 25 districts, respectively. Yet data from the NRL and suspected human and animal rabies cases as declared by the VHOs did not correlate well. The comparison of NRL and VHOs sources showed that in 37 districts VHOs had heard of or observed animal rabies cases although no sample had been sent to or confirmed by the NRL. Furthermore, in 7 districts, VHOs had heard of or observed human rabies cases although no sample had been sent to the NRL (Supplementary Figures 2, 3) (9).

### 3.5 Socio-cultural aspects of dog ownership in Madagascar

According to VHOs, almost everywhere in Madagascar, people keep a dog in/around their home for security reasons (89/90 VHOs). In addition, VHOs indicated that most Malagasy, especially in rural communities, are not inclined or cannot afford to spend money on treating or vaccinating their dogs. A VHO stated that many dog owners reject the idea of vaccinating dogs against rabies even if it is offered free of charge and 67/90 VHOs said that the local communities think that dogs are insignificant animals. As an example, one VHO said: “People keep dogs in their backyards but do not really care for them … they do not care about treating a dog … Even where there is a free vaccination campaign, there are still a lot of people who do not care and do not want to vaccinate their dogs.” In addition, according to 20 VHOs working in the regions of Melaky, Menabe, Atsimo Andrefana, Atsimo Atsinanana, Vatovavy Fitovinany, Androy and certain areas of Fianarantsoa, the local communities consider dogs as taboo (“fady” in Malagasy) (Figures 1, 2) (12, 13). In these regions, even though dogs are present, they are rejected and touching or caring for them is insulting and goes against cultural beliefs. Even burying a dog would spoil the land (14).

### 3.6 Challenges in animal rabies surveillance

#### 3.6.1 Notification of a suspected rabid dog to the VHOs by the population

The 80 VHOs who responded that they had heard/observed cases of rabies in their area said that once people have identified a biting dog or a suspected rabid dog, they kill it directly and dump the carcass into waterways (3 VHOs) or somewhere on the ground without notifying the VHO (80 VHOs). As a result, many suspected rabies cases go unreported and unidentified by the latter. However, the VHOs are sometimes contacted when a human is bitten in an area remote from an ARTC.

#### 3.6.2 Sample collection by the VHOs

Three main problems were identified as potentially hindering animal rabies surveillance in terms of sample collection. Firstly, 35% of interviewed VHOs raised the problem of a lack of knowledge of procedures for collecting a sample for rabies diagnosis. Then, 19% of them mentioned a biosafety problem linked to the lack of personal protective equipment. Finally, 87% of VHOs reported a problem related to the lack of equipment for packing samples, in particular for maintaining the cold chain.

#### 3.6.3 Sending samples to the NRL

Regarding sending a sample from a suspected rabid case to the NRL, some VHOs agreed that there was a huge problem of accessibility. Eighteen (20%) of them mentioned the lack of roads and bridges, with some remote areas accessible only by 4×4 vehicles, and/or the absence of an official postal or courier system, which all contribute to the fact that very few samples are taken and sent to the VHOs in first instance. Then, if a VHO receives a sample for rabies diagnosis, they mentioned that he/she will face the same problems.

#### 3.6.4 Financial constraints

The 80 VHOs who reported the presence of rabies in their area described a major funding problem for rabies surveillance, including notification of rabies cases. The statement “who is going to pay the costs of rabies surveillance” was made by 66% (53/80) of VHOs during the interviews. In addition, 82% of them stated that owners were unwilling and 9% that they were unable to pay the costs of sampling, packaging and postage, and that they, the VHOs, needed to be subsidized given that they were paying the expenses out of their pocket.

## 4 Discussion

The main income generating activities of the VHOs, are livestock vaccination and meat hygiene. According to them, rabies is present in most regions of Madagascar. While it affects livestock, the disease remains marginal to their activities. However, canine rabies-related activities, such as identifying suspected cases, taking and sending samples for diagnosis and notification, represent an expense rather than an income-generating activity, due to a lack of funding. They are therefore not very keen to be part of the rabies surveillance system.

During interviews, VHOs reported having heard of a total of nine cases of human rabies during their VHO’s activity (Figure 1). In comparison, laboratory surveillance reported 36 laboratory-confirmed human cases over the past 10 years in 18 districts (Figure 3). These 18 districts are closer to the capital and clinicians are likely better informed on their role in notification and collaborate more closely with the NRL. Comparing NRL and VHO sources showed that, VHOs had heard of or observed animal rabies cases in 37 districts and human rabies cases in 7 districts without any samples having been confirmed by the NRL. These are districts in the periphery, where the submission of samples to the NRL face more challenges. The contrary occurred in 9 districts, where VHOs were not aware of all NRL results. These figures demonstrate that VHOs are not systematically informed of human and animal rabies cases as required by the organizational scheme for rabies surveillance and that communication between the various actors involved in rabies surveillance clearly needs to be improved. It also suggests that the difference between the effective absence of disease and the absence of notification of a health event needs to be urgently assimilated by many actors in Madagascar, and that, for under-reported diseases such as rabies, several sources of information need to be completed, combined and crossed until concordant figures are obtained.

In Madagascar, rabies is very likely under-reported given that according to the VHOs (i) suspected rabies cases are seldom reported to the VHOs by the population, (ii) samples are rarely taken from suspected cases and (iii) if taken, their shipment to the NRL is very difficult due to logistical and financial issues. These are likely the main reasons why regions far from the capital Antananarivo, have only sent 0–2 samples of suspected rabies cases annually over the last 10 years (9). Underreporting for human cases is also reported by Rajeev et al., (7) who estimated 960 (95% Prediction Intervals (PI): 790–1,120) human deaths from rabies annually.

Dog owners are required to submit the biting animal for veterinary observation (Decree n°3483/99 – Ministry of Agriculture and Livestock). As there is no financially-supported surveillance program, owners are officially responsible for assuming the financial burden in the event of a dog bite. This is probably another reason why suspected rabies cases go unreported, as people might shy away from this responsibility for fear of having to pay for both the medical care of those bitten (PEP, transport, medication) and the laboratory diagnosis in case of suspected rabies (mainly transport costs) (this info had been communicated to one of the authors during stakeholder workshops on rabies prevention and control). Furthermore, even for wealthy families, bringing a dog to a veterinarian or getting a veterinarian to visit a biting dog can be extremely difficult, given the very limited number of veterinarians, especially in rural areas.

The stakeholders in charge of surveillance must improve the surveillance system and increase the budget to cover the costs of animal observation, sampling and shipping. Stakeholders at all levels of surveillance should be trained in basic surveillance concepts (including technical workshops on sampling and biosecurity), prevention and control of zoonotic infectious diseases, and the “One Health” approach. Especially human doctors need to be informed on their important role in the rabies notification system. It should be noted that most veterinarians are not vaccinated against rabies (authors personal comment).

Educational programs should target “responsible dog ownership,” which could improve dog care and vaccination coverage. However, if people are suffering from poverty, as is the case in most parts of Madagascar, sheer survival is the main concern. Preventive measures concerning dogs will therefore have to be 100% subsidized. While many VHOs mentioned in the present study that dog vaccination was not tolerated by the population, Filla et al. (4) found in Moramanga in 2018 (where dogs are not taboo) that 60% of people agreed to vaccinate their dogs if the costs were covered.

During a Knowledge, Attitude and Practices (KAP) survey in Moramanga, 28 bitten people were interviewed. It was reported that only five dogs had been killed, of which four had bitten their owners (5). Rajeev et al. (8) showed that the percentage of biting dogs, which were killed was 1% in dogs classified as non-cases, 3.7% in dogs of unknown rabies status, 33.8% in probable rabies cases and 73% in confirmed rabies cases. Hence, the statement of the VHOs that most biting dogs are killed is not confirmed by the published data. The decision whether to kill a biting dog probably depends on several factors, such as the presence of someone who can kill the dog, the likelihood a dog is rabid or not (including whether the dog exhibited clinical signs of rabies), whether the bite was provoked or not, whether the dog had bitten other people or animals and whether it was vaccinated or not, whether the dog has an owner or not, and the social impact of the decision within the community.

The fear of feral dogs (feral dogs without owners) and their preponderant role in rabies transmission are repeatedly reported (5). However, firstly, often feral dogs are in reality free roaming dogs with unidentified owners, and secondly, when tracing biting dogs, most bites in Madagascar are not due to feral dogs [(5), CTAR data, personal communications].

Given that certain opinions held by the VHOs were not confirmed by field studies carried out in the country, such as the fact that most biting dogs are killed or the preponderant role of stray dogs compared to owned dogs in rabies transmission, it would be interesting to conduct studies on these topics and communicate these field study results to both veterinary and public health officers, VHOs and veterinary students.

Many low-income countries face problems of budget, infrastructure and a low coverage of veterinary services. In Madagascar, socio-cultural beliefs toward dogs in 14 districts, mainly in the Western, Southern and Eastern coastal regions, where they are “fady” (taboo), represent an additional challenge to rabies surveillance and control. Apparently, the compliance of the local communities to rabies surveillance and control might be difficult and handling dogs in any way creates tensions between the authorities and local communities. As a result, VHOs in these regions are not active in rabies prevention and control. Consequently, in the opinion of many VHOs, implementing a mass vaccination or sterilization campaign would be impossible in these areas, as traditional village authorities would adamantly be opposed to such measures. The following statement from a VHO illustrates the situation: “the society will reject you if you take care of a dog or touch it … even burying a dog is forbidden here, it is a taboo …, vaccination is impossible. It is a big problem here.”

The fear of veterinarians of being rejected if they treat or vaccinate dogs in areas where dogs are taboo was confirmed by a KAP study conducted in Menabe 2020–2021 (12, 13). In this context, to carry out vaccination campaigns in regions where dogs are “fady,” the temporary mobilization of veterinarians from outside these regions, with the prior agreement of local authorities, could be a solution. In any case, the first step is to ensure that the population will accept dog vaccination. Further, we recommend conducting studies on the “implementability,” safety and efficacy of the use of controlled oral vaccination in food baits, which could be a way to avoid handling these dogs which are not used to being touched (15). However, these live vaccines hidden in an edible bait are likely to encounter resistance from a population many of whom suffer from hunger, and who might not understand why “dogs are fed” while children are malnourished. Whether this assumption is correct and which communication and participatory strategies would be needed to improve the acceptance of the population (if the “oral vaccine strategy” was a control option) warrant a qualitative research approach. An interesting One Health approach would be a collaboration of international and national organizations and NGOs involved in nutrition programs with those organizing a vaccination campaign or combining vaccination of children (Polio, Measles etc.) with a rabies vaccination campaign of dogs (16).

The KAP survey on rabies, conducted in the community of Moramanga, showed that while knowledge of the main hosts, transmission routes, symptoms and outcomes was high, knowledge of the existence of ARTCs, the usefulness and availability of PEP, and the need to confine and observe biting dogs was dramatically low (5). Therefore, it is important to inform communities about what to do after a dog bite (such as washing the wound with water and soap for at least 15 min, slow the bleeding, and look for PEP) and why dog vaccination is crucial to rabies elimination. The KAP study conducted in the Menabe Region demonstrated that an “awareness approach” can consequently improve the communities KAP regarding rabies (17).

Improving rabies surveillance is a real challenge, as most problems and challenges are poverty-related. Yet the path to rabies elimination has been thoroughly documented (18–20) and several authors have synthetized lessons learned to help countries willing to embark on this path (21–23). So far, in Madagascar, efforts have focused on eliminating human deaths due to rabies thanks to the privately-funded support of IPM, which offers PEP for free to the ARTC network (24). PEP is highly effective in preventing rabies deaths in humans, but it is well-known that only mass vaccination of dogs can lead to the elimination of dog-transmitted rabies. Although research has proven the ineffectiveness of dogs culling in rabies control (25), the current official recommendation in Madagascar is still based on dog culling (Decree 3482/99 – Ministry of Agriculture and Livestock).

Mass vaccination of dogs has been shown to be very cost-effective, particularly if carried out with a well-tailored One Health communication (26, 27). Madagascar has recently received dog vaccines and has begun mass vaccination in two regions. We therefore recommend pursuing these efforts and focusing on free mass vaccination of dogs in combination with awareness campaigns. To properly plan vaccination campaigns, it is recommended to estimate the turnover rate of the local dog population to adapt the frequency of dog vaccination. Further, to collect epidemiological data from active rabies surveillance (through sentinel sites?) to identify high-incidence areas in densely-populated zones to prioritize the locations for vaccination campaigns given limited funds. Most importantly, politicians and stakeholders need to be convinced of the importance of rabies prevention and control. This is a challenge in a country facing many poverty-related problems, with a wide range of communicable and non-communicable diseases. While the prevention and control of malaria, tuberculosis and plague has received much attention and funding, neglected diseases such as rabies require more attention, as the burden is high, especially in the underprivileged populations, who often remain forgotten (28).

### 4.1 Limitations

This mixed-methods study represents the opinions of 90 VHOs from different regions of Madagascar. By interviewing 60% (90/150) of all VHOs, a large variety of professional profiles was included, and all regions of Madagascar were represented. The opinions of the VHOs, which are based on personal experiences and convictions, may not represent the opinions of all. Nor do they represent the opinions of other stakeholders in the rabies surveillance system. Veterinarians who recently started working as VHOs (2 years) may have less knowledge on the rabies situation, in comparison to those who have been longer established in their working area (31 years). Nevertheless, the fact that all 22 regions of Madagascar were represented indicates a relatively good geographical coverage of the study. The second part of the interview was an open discussion. The opinions/answers of the VHOs were categorized in a thematic grid and presented in the results section. However, not all VHOs mentioned the same topics. For example, 18/80 VHOs mentioned the negligence/unawareness of the danger of dog bites as a reason for the occurrence of human rabies cases. These figures do not mean that the other 62 VHOs would not have made the same statement. They simply did not mention it during the open discussion. The advantage of the open discussion is that it allows to be informed of the opinion of the VHOs without influencing them, but the disadvantage of this non-systematic approach is the lack of representativeness.

### 4.2 Conclusion

This study shows that rabies cases are frequently observed by VHOs in the field, in all the 22 regions of the country, but that several obstacles hinder rabies surveillance, leading to under-reporting of cases. The main barrier to surveillance is financial, as noted by all the VHOs interviewed. Lack of funds to access suspected animals, collect and package samples, comply with biosecurity and cold chain measures, and ship samples are major obstacles to notification. The lack of funds has also a negative impact on dog owners’ willingness to report a bite and follow procedures, as they are often reluctant -or unable- to cover the associated costs. The second obstacle identified by VHOs is socio-cultural. In many large coastal regions of the island dogs are taboo and VHOs fear rejection by the community if they take care of dogs. Moreover, the lack of community awareness of rabies and PEP was mentioned several times. Finally, the poor correlation between rabies cases confirmed by the NRL and rabies cases reported by the VHOs underlines the need to improve the information and communication within the surveillance network. In this context, while the general population needs to be informed about the rabies situation in Madagascar, that vaccination is crucial to control this disease and how to proceed in the event of a bite, veterinarians and decision-makers need to be fully aware of certain epidemiological concepts to understand the usefulness of an evidence-based approach to rabies surveillance, prevention and control. Stakeholder workshops to develop a program for the improvement of rabies surveillance in Madagascar using a participatory approach is highly recommended. For their part, politicians need to be persuaded of the importance and necessity of funding to eliminate rabies in Madagascar. The adoption, in early 2023, of a national strategic plan for rabies control is a first step in this direction.

## Data availability statement

The data supporting the conclusions of this article will be made available by the authors, without undue reservation.

## Ethics statement

Ethical review and approval was not required for the study on human participants in accordance with the local legislation and institutional requirements. Written informed consent from the participants was not required to participate in this study in accordance with the national legislation and the institutional requirements.

## Author contributions

AD: Conceptualization, Formal analysis, Methodology, Supervision, Writing – original draft, Writing – review & editing. MV: Conceptualization, Formal analysis, Methodology, Writing – original draft. HG: Conceptualization, Formal analysis, Methodology, Visualization, Writing – original draft, Writing – review & editing. NiR: Supervision, Writing – review & editing. MH: Investigation, Writing – review & editing. NA: Investigation, Writing – review & editing. NaR: Supervision, Writing – review & editing. MA: Supervision, Writing – review & editing. PD: Supervision, Writing – review & editing. DK: Conceptualization, Writing – review & editing. VL: Methodology, Writing – review & editing. SF: Conceptualization, Formal analysis, Investigation, Methodology, Supervision, Visualization, Writing – original draft, Writing – review & editing.

## Footnotes

^1^https://en.wikipedia.org/wiki/Antemoro_people, accessed 11.01.2024.
