# Supplementary material for: Challenges of rabies surveillance in Madagascar based on a mixed method survey amongst veterinary health officers
Source: Front Vet Sci. 2024 Feb 28;11:1270547. doi: 10.3389/fvets.2024.1270547 (PMC10938849; doi:10.3389/fvets.2024.1270547)
Supplement: Supplementary file 1 [file Data_Sheet_1.docx]

Oral consent to participate: yes ❒ no ❒

If no, why?

| 1. Investigator’s name: | |
| --- | --- |
| 2. Participants name: | |
| 3. City: | |
| 4. Phone contact: | |
| 5. Date and time of the start of survey: | |
| 6. Type of position:   - Veterinarian health officer ❒ - Sentinel vet ❒ | - Veterinarian❒ - Other ❒ (specify: ) |
| 7. For how many years have you been practicing as a veterinarian? | |
| 8. For how many years have you practiced in this area? | |
| 9. What is the radius of your customer base?   - <5 km ❒ - 5-10 km ❒ - 11-25 km❒ - >25 km❒ | |
| 10. Do you have an assistant to help you handle animals? yes ❒ no❒ | |
| 11. How many livestock technicians work with you in carrying out your tasks? | |
| 12. What types of animals do you treat?   - Poultry: yes ❒ no❒ - Horses: yes ❒ no❒ - Domestic carnivores: yes ❒ no❒ - Fish: yes ❒ no❒ - Pigs: yes ❒ no❒ - Ruminants: yes ❒ no❒ - Other: yes ❒ (specify: ) no❒ | |
| 13. In your practice, do you take care of dogs? yes ❒ no❒ | |
| 13a. If not: why?   - - Personal conviction ❒   - Socio-cultural context❒   - Owners‘ attitudes ❒   - Other, please specify: | |
| 14. Have there been any suspected or confirmed animal rabies cases in your practicing area since you started working? yes ❒ no❒ | |
| 14a. If yes, how often?   - At least once a week ❒ - At least once of a month ❒ - At least once a year ❒ - Other, please specify: | |
| 14b. Which animal species is most frequently suspected of rabies in your working area or location?   - Ruminant ❒ - Dog ❒ - Cat ❒ - Horse ❒ - Pig ❒ - Other, please specify: | |
| 15. Apart from yourself, who are the main actors involved in rabies surveillance and reporting in your area/location?   - Law enforcement officers ❒ - Anti-rabies treatment centers ❒ - Local authorities ❒ | |
| 16. In your opinion, are the main actors involved in rabies surveillance and reporting aware that laboratory diagnosis of rabies is carried out at the Institut Pasteur de Madagascar? yes ❒ no❒ | |
| 17. In your opinion, are the main actors involved in rabies surveillance and reporting (and your technicians) familiar with the procedures to follow in the event of a case or suspicion of rabies? yes ❒ no❒ | |
| 18. In your opinion, do the main actors involved in rabies surveillance and reporting know that if an animal is suspected of being rabid, it must be taken to the vet for observation? yes ❒ no❒ | |
| 19. Have you already worked with other actors involved in rabies surveillance and reporting? yes ❒ no❒ | |
| 19a. If the response is no (Q19), why?   - Distance issue (means of travel, communication…) ❒ - In most cases, the animal suspected of being rabid is dead or killed and then thrown away, so there’s no need to inform you ❒ | |
| 20. If your technicians suspect rabies in a given animal, are you informed prior to handling the suspected rabid animal? yes ❒ no❒ | |
| 21. Why? | |
| 22. What types of symptoms should you look for when diagnosing a suspected rabies case? (Open question) | |
| 23. Are rabid suspected animals more likely stray dogs or owned animals?   - Stray dogs ❒ - Owned animals ❒ | |
| 23a. In the case of rabid dogs with owners, were these dogs vaccinated against rabies?  yes ❒ no❒ | |
| 23aa. If so (yes at 23a), was rabies vaccination up to date? yes ❒ no❒ | |
| 24. What procedure do you follow if rabies is suspected in an animal (including biting animal)?   - Observation ❒ - Euthanasia ❒ - Nothing ❒ - Other ❒ | |
| 24a. If you observe suspect dogs (Q24): for how many days do you observe an animal suspected of being rabid? | |
| 24b: If you observe suspect dogs (Q24): Can you give us a cost range for each observation? | |
| 24c: If you observe suspect dogs (Q24): Where do you do it?   - Office or dedicated area ❒ - At owner’s home ❒ - Other, please specify: | |
| 24d. If you observe suspect dogs (Q24): What proportion of suspected rabid dogs completed the observation phase? | |
| 24e. If you observe suspect dogs (Q24) and if proportion of suspect dogs completing the observation (Q24d) is <100: In your opinion, what could be the cause of not completing the observation period? | |
| 24f. If you observe suspect dogs (Q24): Did any of the animals under observation develop rabies? | |
| 24g. If you observe suspect dogs (Q24): How often do animals under observation develop rabies? | |
| 25h. If an animal has developed rabies, what action should you take? | |
| 26. Do you take samples for lab diagnosis when the animal dies? | |
| 27. What do you think is a major obstacle to carrying out this procedure? | |
| 28. End of interview: | |

The interview is followed by an open discussion and opinions are recorded and sorted by topic in a grid.
